# Microanalysis of video from the operating room: an underused approach to patient safety research

**DOI:** 10.1136/bmjqs-2016-005816

**Published:** 2016-12-09

**Authors:** Jeff Bezemer, Alexandra Cope, Terhi Korkiakangas, Gunther Kress, Ged Murtagh, Sharon-Marie Weldon, Roger Kneebone

**Affiliations:** 1 UCL Institute of Education, University College London, London, UK; 2 University of Leeds Institute of Medical Education, Leeds, UK; 3 Department of Surgery and Cancer, Imperial College London, London, UK

**Keywords:** Communication, Teamwork, Surgery, Social sciences

## Video recording in the operating room

Video recording technologies offer a powerful way to document what happens in clinical areas.^1^ Cameras, and to a lesser extent, microphones, can be found in a growing number of modern operating rooms in the USA, UK and other parts of the world. While they could be used to create a detailed record of what happens in and around the operating table, this is still rarely being done; the vast majority of operations are still only documented in written operation notes. When operations *are* being recorded, it is primarily for *educational* purposes: for instance, to broadcast a live feed of a surgical demonstration to a remote audience; to provide an ‘adjunct’ to live observation;^2^ to collect authentic footage for edited, instructional videos on a surgical technique or procedure; to facilitate video enhanced debriefing and coaching; or to formally assess surgical skills.

Recently, Makary *et al*^1^ ^3^ have proposed that video equipment in the operating room could be used as an *auditing* tool. They also argue that making video recording a routine occurrence would improve performance and make surgical care more transparent. They propose that a video archive of operations could prove useful for surgeons preparing to operate on a patient who had been operated on before—to check the anatomy and density of adhesions for example. It has also been suggested that when used routinely, video could be used to investigate adverse events.^4–6^

What has gone relatively unnoticed in these recent discussions about the potential of video in the operating room is the possibilities it opens up for empirical *research*. While videos can only provide a partial representation of what happened and are always open to interpretation, they do provide a relatively objective, shareable point of reference. Using video, clinical events can be looked at from different angles. In suitably equipped operating rooms, the laparoscopic camera and light handle cameras provide a detailed view of the operative field, while wall-mounted cameras capture the entire multiprofessional team in action. In-built microphones and wireless microphones worn by members of the team can be used to record verbal communications.

Thus, video recording has now become a relatively cheap, accessible yet powerful method for *collecting* live data. Such data can provide alternative or complementary data to that gained through interviews, where team members provide a retrospective account of events based on their memory of what took place. Video enables different team members, external observers and analysts to review segments of an operation repeatedly, zooming in and slowing down where relevant, so as to verify each other's judgements and interpretations and develop a shared understanding of what took place.

However, using video as data for patient safety research demands apt *methodological frameworks*. One such framework was developed by Iedema *et al*.^7–9^ Their video reflexive ethnographic (VRE) approach invites health professionals to jointly watch and interpret selected video fragments and reflect on their implications for patient safety. For instance, participants have identified new approaches to infection control, to improving intensive care unit ward rounds and to the design of local clinical work spaces. VRE offers a way of innovating and improving from within and from bottom up, inviting people to learn through reflection on their behaviours. As far as we are aware, this approach has not yet been applied to the operating room.

## Microanalysis of video recordings

The methodological framework we introduce here combines elements from sociolinguistics, conversation analysis and social semiotics. It brings new insights into clinical practice by detailed, systematic *microanalysis* conducted by a multiprofessional team of analysts using specialist software. The framework offers tools for systematically describing, analysing and, ultimately, predicting behavioural patterns. It is unique in that it operates on a ‘microscopic’ level, highlighting human actions—spoken utterances, gestures, gaze shifts—involved in clinical events and illuminating the ways in which they are interwoven, taking full advantage of the fine detail offered by the video record.

The framework has been used in a range of studies on (clinical) work environments, including surgery, anaesthesia, dentistry and emergency medicine.^10–12^ Yet with the exception of a handful of studies,^13^ ^14^ that work was conducted by and for social scientists. Our own research team is multiprofessional and includes healthcare practitioners (‘insiders’ in surgery and perioperative nursing), working in close collaboration with social scientists. Our primary aim is to contribute to knowledge and understanding of quality and safety by applying the insights gained from microanalytical procedures just described.

Our approach rests on the tradition of naturalistic inquiry, where fieldwork is conducted to observe behaviour in actual clinical settings rather than experimental controlled settings.^15^ Video-based technologies are used to identify and attend to details of social interaction that are not adequately captured by other data collection techniques. This allows aspects of clinical work that are too small scale and ephemeral to be noticed in real time. By replaying recordings multiple times, nuances of speech and tiny gestures which would otherwise be overlooked can be documented and analysed. Such fine-grained analysis discloses aspects of team work—manners of talking, acting and reasoning—that participants themselves are often unaware of (and are therefore unlikely to notice, let alone reflect on).

We adopted this approach in a series of studies, investigating communication, situation awareness, teamwork, clinical decision making and surgical education in the operating rooms of a major hospital in London. Ethical approval was granted by the UK National Health Service Research Ethics Committee (ref nr 10/H0712/1). From 2010 and 2013, we audio and video recorded 42 operations, covering 66 hours of operating time. Procedures included a mix of general surgical, upper gastrointestinal, colorectal and bariatric procedures. These ranged from simple skin lesion excisions under local anaesthetic to major cancer operations taking an entire day. A wireless microphone was worn by one of the surgeons. Video recordings were collected using a camera built into a light handle, camcorders mounted on tripods and (where relevant) the laparoscope. All participants were aware that data were being collected to study behaviours in the operating room but no evidence was found in the video data that participants responded adversely to the presence of cameras. Key to the analytical process was the repeated inspection, transcription and coding of verbal and non-verbal communication to produce a detailed time log (table 1).

**Table 1:** Fragment of a detailed log

01.242	Surgeon	‘Scissors please’	Verbal request
03.551	Scrub nurse	‘Dissecting scissors?’	Clarification request
05.321	Surgeon	‘No’	Clarification
07.040	Scrub nurse	Offers stitch scissors	Offering
07.748	Surgeon	Takes scissors from nurse	Acceptance

Professional transcription and annotation software is available to support this logging and coding process (eg, Noldus Observer). Quality control in our studies was achieved by having multiple researchers code a sample of the video recordings and compare and discuss any differences. In this way, transcribers developed a shared understanding of the codes used.

When systematically coded, logs can be used for three different analytical purposes.

First, they can be used to count the *prevalence of certain behaviours* within the sample. For instance, in one study^16^ we marked the instances when surgeons make verbal requests for instruments and the occasions when scrub nurses ask surgeons to clarify the request they made, and *measured* the time lapse between the completion of the verbal request, the clarification and the passing of the required instrument by the nurse (as illustrated in table 1). The prevalence of repeated requests and time to complete instrument requests are examples of relatively objective indicators of the level of communicative effort needed to achieve the tasks at hand. Non-verbal offerings, for example, when a nurse anticipates what the surgeon will need and offers an instrument without a direct request from the surgeon, can be logged in a similar fashion. The surgeon's response to such offerings can then be coded as acceptance or rejection of the instrument, providing more insight in the team's performance and situation awareness.

In another study,^17^ we used the logs to gain insight in clinical reasoning and decision making by consultant surgeons and surgical trainees. Looking at laparoscopic cholecystectomy, we found significant variation in the amount of time dedicated to discussing anatomy in Calot's triangle (1%–60% of operating time). We also investigated differences and shifts in the level of certainty achieved about the identification of anatomical structures by systematically mapping linguistic markers of ‘modality’, such as ‘clearly is’, ‘might be’ and so on. This provided insight into how surgical trainees learn in the operating room, what safeguards are present and how these relate to official norms. For instance, surgical textbooks point to the need to create a ‘safety window’ before clipping the cystic duct. Yet only through participation in surgical practice can surgical trainees learn what, in that local setting, counts as a safety window and what constitutes an acceptable degree of uncertainty. Microanalysis can help make such tacit safety norms explicit.

Second, video logs can be used to explore the likely *effects of certain behaviours*. For instance, we explored how a team member's use of language affects whether and how colleagues are likely to respond. We found that during laparoscopic cholecystectomies surgeons involve their assistants in different ways in the decision to clip and cut the cystic duct.^18^ Some would ask, ‘Are you happy?’, others ‘What do you think?’ The closed question format of the former (the most common form in our sample) always led to the assistant affirming they were happy. The open question format of the latter, which occurred relatively infrequently, invited the assistant to make a proposal for action (‘I think you have to clip that’). Analysing many different examples of this kind, we have found that the effectiveness of different strategies is relative to what they are designed to achieve. For example, if the aim is merely to garner confirmation and/or to implicate assistants in a decision, then a closed format is the most apt form. However, if the aim is to create an additional ‘defence wall’ against misidentification, minimising barriers for the assistant to speak up, then an open format seems preferable. These findings indicate that something as apparently straightforward as the linguistic formats people choose for communicating with their team may influence safety and patient outcomes.

In another study,^19^ one of the authors (TK) explored how team members responded to calls for time outs to do the WHO Surgical Safety checklist. By systematically mapping the timing of each call (time lapse between patient transferred to operating table and beginning of time out), the means by which the calls were realised (loudness of voice, gaze direction) and the differential responses triggered among the members of the team, it was found that the gathering or ‘mobilisation’ of teams is a key factor in maximising participation from all members of the team in time outs. The study also generated a proposal about when and how calls for time out should be made to maximise inclusion of team members.

Third, video logs can be used to explore the *association of certain behaviours* with contextual factors. For instance, we measured the loudness of music being played in the operating room, correlating this with the frequency of repeated requests for the same instrument, indicating that the original request had not resulted in the correct instrument being transferred. We found repeated requests were five times more likely to be made where music was played than where it was not.^20^ These findings suggest that communication can be compromised when loud music is played during surgery.

## Using video in engagement and simulation-based training

As well as detailed analyses such as those outlined above, we have used our video recordings to involve surgeons, nurses and other health professionals with our research. Selected clips were also used to involve specialist social science audiences and the general public directly in the research and in discussions around patient safety.

We have conducted a number of events using anonymised video clips to (a) provide an empirical point of reference for and promote discussion about surgical practices, (b) present key findings from our research on behaviour and communication in the operating room and (c) verify the extent to which these findings resonate with our audiences.

For instance, we showed video clips illustrating the loudness of music sometimes played at our research site to highlight occasional challenges experienced by nurses in hearing what surgeons were requesting. The clips provided evidence of the impact that music can have on surgical teams, and prompted discussions exposing a range of views among clinicians and publics around the pros and cons of playing music during surgery.

We have also used recordings to develop Video-Supported Simulation for Interactions in the Operating Room.^21^ In one-day training events for operating room teams, we use ‘authentic’ scenarios based on documented events. In the debriefing that follows each simulation, we show the anonymised clip upon which the scenario was based, using this to trigger reflection by the team and identify action points for change.

## Future directions

The examples outlined above indicate that detailed analysis of video holds significant potential for improving patient safety. Further use of video research will benefit from a *large video database*. Before data can be collected on such a scale, there are ethical and medicolegal issues to be addressed.^22^ ^23^ Staff, patients and other potential users of video data must agree on who can access what video data and for what purposes. The circumstances under which the data might need to be released (eg, in cases of litigation) need to be clearly stipulated. While the collection of video data is a relatively complex process, once a video corpus has been created it can be used to generate and test multiple hypotheses across different studies. There are obvious economies of scale in research teams from different institutions working together to build such a shared video corpus.

Video recording within surgery offers rich possibilities for *mixed methods research*. One potential area is to explore correlations between the detailed breakdowns of observed behaviour illustrated in this viewpoint and conventional global ratings based on existing observation tools. This could help make explicit exactly what raters believe to be indicators of ‘good’ practice, specifying relatively vague descriptors in observation tools, such as ‘the communication was clear’. These specifications could be used to develop a specialist language for talking about and reflecting on clinical work. Associations could also be made between the occurrence of patterns of communication and patient outcomes.

Video recordings can be usefully supplemented with *data collected using other digital technologies*. Eye-tracking glasses can be used to gather detailed information about team members' focus of attention,^24^ while hand movements and whole-body movements in the operating room can be tracked using motion capture sensors.^25^ ^26^ Such information could be incorporated in logs such as the example above, further advancing the mapping of team behaviours in real time. At the moment motion tracking technologies are expensive, and using them to document real operations requires significant effort by both researchers/technicians and participating clinicians.

Possibilities for exploring behaviours and their relations to the room environment and patient outcomes are greatly expanded with the introduction of integrated systems or ‘black boxes’.^27^ ^28^ Such technologies collect a wide range of digital data from a range of devices and sensors in the operating room alongside audio and video recordings, providing information about the patient's physiology (such as vital signs) and the room environment (eg, room temperature, noise level, air quality, frequency of door openings). These systems are likely to significantly enhance our understanding of the complex web of factors at play at any one time in an operating room, and their effects on the patient.

Though the focus of this paper is the operating room, we believe that opportunities for microanalysis of video exist across different areas of healthcare. Of course video has been used by social scientists to study clinical practice for decades, and there is significant methodological expertise in this area. Yet to date, little video research of the kind presented here, with its detailed analysis of behaviour and interaction, has directly addressed issues around patient safety; it has, by and large, been designed for social scientists rather than for health professions. It is time for health professionals and patients to take advantage of this approach, and for existing methodological expertise to be unlocked, applied and advanced for the benefit of all.
